# Application of magneto-luminescent gold nanoclusters with microfluidic systems to the determination analysis of tetracyclines

**DOI:** 10.1007/s00604-025-07405-5

**Published:** 2025-07-31

**Authors:** Javier Palma-Roldán, Vanesa Román-Pizarro, Miguel Ángel García-Granados, Juan Manuel Fernández-Romero, Ángela Écija-Arenas

**Affiliations:** Departamento de Química Analítica, Instituto Químico Para La Energía y El Medioambiente (IQUEMA), Universidad de Córdoba, Edificio Anexo “Marie Curie”, Campus de Rabanales, 14071 Córdoba, Spain

**Keywords:** Magnetic gold nanoclusters (AuMNCs), Microfluidic systems, Fluorometric detection, Tetracycline determination, Environmental samples

## Abstract

A microfluidic analytical system based on magnetically retained nanomaterials has been developed using fluorometric detection. This system utilizes magnetic gold nanoclusters (AuMNCs) to determine total tetracyclines using three of them as model—chlortetracycline (CTC), doxycycline (DC), and oxytetracycline (OTC)—in water samples by monitoring the change in the luminescent signal produced upon interaction. AuMNCs were synthesized by adding preformed magnetic nanoparticles (MNPs) to bovine serum albumin (BSA) as stabilizer and ascorbic acid as a reducing agent. Their magnetic properties enable their retention in the reaction/detection zone of the microfluidic system. The reactor was integrated into the optical pathway of a conventional spectrofluorometer with a 3D-printed device to align the excitation beam with the microchannel. The instrumental signal was obtained at a 22.5° angle from the excitation beam. Key parameters were studied, including pH (optimal at 11 with carbonate buffer), flow rate (40 µL·min⁻^1^), and AuMNCs injection volume (10 µL). The analytical signal was based on the fluorescence intensity ratio between the emission of the AuMNCs (650 nm) and the AuMNCs-tetracycline complexes (425 or 510 nm). Limits of detection of 0.41 µmol·L⁻^1^ for CTC, 0.70 µmol·L⁻^1^ for DC, and 0.61 µmol·L⁻^1^ for OTC, with relative standard deviations (RSD) below 6.2%, were achieved. Compared with non-retained AuMNCs, the magnetically retained system showed improved results. The system was applied to determine different tetracyclines in water samples, with recoveries in spiked river water samples from 86 to 111%. This approach offers a simple, label-free, and versatile alternative with potential for future development into multi-analyte sensing platforms or applications in complex matrices.

**Graphical Abstract:**

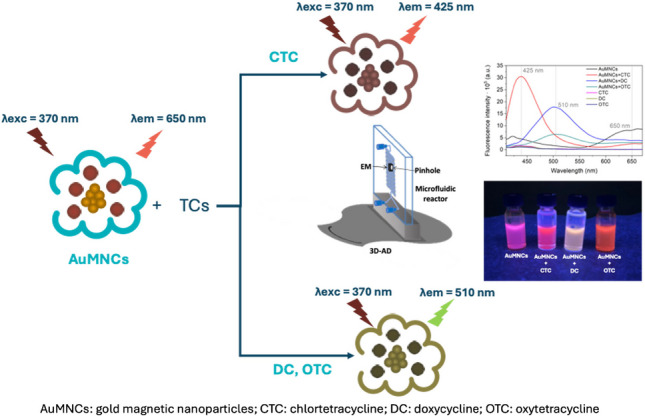

**Supplementary Information:**

The online version contains supplementary material available at 10.1007/s00604-025-07405-5.

## Introduction

During the last decades, science has evolved towards miniaturization, and even smaller-scale nanomaterials (NMs) have been designed. During the development of this research work, a type of NMs, such as metallic nanoclusters (MeNCs), has been synthesized. MeNCs are structures composed of dozens of metallic atoms stabilized by molecules, which have a size ranging from 1 to 3 nm. The electronic interactions between the organized and stabilized atoms present unique optical, electronic, chemical, and magnetic characteristics that allow their wide application [1]. Among these, it is worth highlighting their high surface-to-volume ratio, small size, surface functionalization capacity, and spectroscopic properties, including possible luminescent emission and surface plasmon resonance. They also have disadvantages, such as short lifetimes, forming larger nanoparticles without unique size properties, or exhibiting aggregation potential [2].

MeNCs are synthesized through a bottom-up synthesis procedure, which begins with metallic precursors, the most common being Au^3+^, Ag^+^, and Cu^2+^. Different synthesis models are employed in this case, specifically direct synthesis, where the metallic ion is exposed together with the stabilizing molecule to achieve a combined effect that encompasses both the reduction and growth stages, thereby limiting the shape and size of the resulting material and achieving stabilization. The stabilizing molecule can be a nucleic acid, protein, peptide, or oligonucleotide. In this case, gold nanoclusters (AuNCs) have been synthesized using bovine serum albumin (BSA) as a stabilizing molecule. The process is based on the reducing capacity of the tyrosine residues contained in the BSA protein once it interacts with the Au^3+^ ions to achieve the nucleation of metallic gold and the association complex in a basic medium, in addition to the stabilizing function due to its three-dimensional structure and the location of its amino acids allowing it to capture, reduce and stabilize gold atoms [3–5].

AuNCs exhibit advantages such as photostability, long lifetime, high photoluminescence, high quantum yield, and ease of tuning the emission wavelength depending on their size since the emitted fluorescence will depend on the oxidation state of gold, the size of the formed core, the interaction between ligands and the surface of the metallic core [6–8]. In addition, compared to conventional fluorophores, they have a smaller size, low toxicity, low photobleaching, and good biocompatibility. They also have high catalytic activity and chemical stability. The mentioned particularities enable AuNCs to be utilized in various fields, including electrocatalysts, bioimaging agents, drug delivery, disease treatment, and chemical sensors [9].

The aim is to provide magnetic properties to the synthesized AuNCs, obtaining magnetic nanoclusters (AuMNCs). Magnetic nanoclusters (MNCs) possess the properties of nanoclusters in addition to the ability to retain magnetic species. These magnetic properties are acquired by adding magnetic nanoparticles (MNPs) to synthesize nanoclusters. This synthesis can follow various procedures. On the one hand, synthesizing MNPs that are subsequently adsorbed to the AuNCs with the stabilizing protein makes them stable at different pH [10]. On the other hand, the synthesis of MNPs and NCs can be carried out simultaneously through a solvothermal reaction with a stabilizing agent, resulting in MNCs with superparamagnetic properties and high magnetization values [11]. MNCs have a wide variety of functions, including the ability to immobilize samples using a magnet, which saves resources and reduces the consumption of nanoclusters without compromising sensitivity or precision in the method. MNCs can be synthesized following various procedures.

The interaction of synthesized AuMNCs with a group of molecules, tetracyclines, has been studied. Tetracyclines are among the most widely used antibiotics. Their study is interesting because it highlights the serious environmental problems caused by many of these products, which are often emitted after use. They have a very hydrophilic character and low volatility, which causes significant persistence, especially in aquatic environments. These antibiotic residues can lead to the development of antibiotic resistance, which can have adverse effects on human health. Additionally, their effective elimination is practically impossible for most wastewater treatment plants [12]. Therefore, it is helpful to have methods for determining these antibiotics to know their present levels. Determining tetracyclines in various areas requires complex instrumentation, with a high sample preparation and significant time consumption, such as liquid chromatography (HPLC) [13]. The method proposed in this research represents a methodological novelty. This will involve the application of the previously synthesized AuMNCs using a microfluidic system coupled to a fluorimetric detector to monitor the luminescent signal caused by the interaction between the AuMNCs luminescent complex and the tetracycline residues. Some microfluidic systems have been developed using magnetic or gold nanomaterials, but not gold magnetic nanoclusters joined [14–17]. The advantage of using microfluidic systems lies in the ability to work with small volumes of reagents and samples, their cost-effectiveness, and the speed of analysis. The basis of this method is the modifying effect that the analyte can exert on the involved protein, thereby modifying the emission bands of the AuMNCs [18]. Different methodologies have been described based on the interaction between AuNCs bound to BSA and various tetracyclines, monitoring the spectral modifications of these formed complexes [19]. This work presents a methodology for determining total tetracyclines using three of them as model (chlortetracycline, oxytetracycline, and doxycycline) in previously treated aqueous samples.

As mentioned, the interaction between synthesized AuMNCs and tetracyclines has been carried out using a microfluidic scale system. Integrating and adapting the analytical process stages to a microfluidic scale requires significant effort in terms of miniaturization and fluid adaptation. The most significant advance is based on the shift from macro-scale to micrometric-scale flow systems and the corresponding changes in the behavior of physical, chemical, and physicochemical properties [20]. On the micrometric scale, fluid physics and physicochemical interactions between the reaction and its environment occur in a laminar flow context, making it challenging to mix reagents effectively. However, systems under this scale present advantages such as lower sample and reagent consumption, higher sampling frequency, portability, and shorter analysis time [21].

In the case of this research, since luminescence measurements of AuMNCs are to be carried out, the microfluidic system will be coupled with a spectrofluorometer. The reaction zone of the microfluidic system will be joined with the detection zone since, taking advantage of the magnetic characteristics of the material, it will be magnetically retained with an electromagnet in said zone, and the aim will be to visualize the change in the fluorescent signal of AuMNCs based on the interaction with the tetracycline molecules. Integration of the reaction/detection zone of the microfluidic system with the luminescent detection system can be done in various ways using, in this case, the integration of the microfluidic system in the conventional detector from elements that enable the focusing of the channel of the microfluidic system in the light beam of the detector, in which the reaction/detection stage will be developed. This choice requires incorporating an alignment device manufactured using the 3D printing methodology to position the chip holder containing the chip within the microfluidic reactor in the sample compartment of the spectrofluorometer, thereby measuring the instrumental signals in the detector. This placement enables the integration of the microfluidic system without compromising the sensitivity characteristics of a conventional spectrofluorometer [22].

Integrating luminescent nanocrystals (NCs) with magnetic properties into microfluidic systems enables the creation of sensitive sensors for detecting and quantifying chemical compounds, thereby combining the advantages of both approaches. These sensors have been applied to the determination of total tetracyclines in aqueous samples based on the change in fluorescent signal that occurs upon the interaction of these antibiotics with the retained NCs.

## Materials and methods

### Materials

All reagents and solutions used during this research were of analytical grade. Sodium hydroxide, disodium phosphate (Na_2_HPO_4_), tris(hydroxymethyl) aminomethane (Tris–HCl), and citric acid were purchased from Merck (Merck Group, Darmstadt, Germany, https://www.merckgroup.com/en). Tetrachloroauric acid (HAuCl_4_), doxycycline hydrochloride (DC), bovine serum albumin (BSA), iron (II) chloride, chlortetracycline hydrochloride (CTC), oxytetracycline hydrochloride (OTC), hydrochloric acid, and ascorbic acid were purchased from Sigma-Aldrich (Sigma-Aldrich Química, Madrid, Spain, https://www.sigmaaldrich.com/). Methanol and ethylenediaminetetraacetic acid (Na_2_EDTA) were purchased from Honeywell (Honeywell, Madrid, Spain, https://lab.honeywell.com/en/fluka). Iron (III) chloride and disodium carbonate (NaHCO_3_) were purchased from Panreac (Panreac Química S.L.U., Barcelona, ​Spain, https://www.itwreagents.com/iberia/es/oficina). The aqueous solutions were prepared from deionized water purified with a Mili-Q system (Millipore, Bedford, MA, USA, www.merckmillipore.com/).

### Apparatus and instruments

A Büchi R-200 Rotavapor (Flawil, Switzerland, http://www.buchi.com) was used to synthesize AuMNCs. For the synthesis of MNPs, an MPW-350R centrifuge (MPW Med. Instrument, Warsaw, Poland, http://www.mpw.pl) with a rotating cooling chamber, an HSL-11199 angular rotor (45°, 12 × 2.2/1.5 ml, max. speed = 18,000 rpm, 24,088 RCF, and rmax = 6.65 cm) was used. For homogenization, an ultrasonic bath was used.

The synthesis of AuMNCs was characterized using the Zetasizer System (Malvern Instruments Worcestershire, United Kingdom; https://www.malvernpanalytical.com/es). The measurements of the developed system were performed with the Horiba Scientific Fluorolog-3P spectrofluorometer (Horiba Scientific, France, www.horiba.com/scientific/). The spectrofluorometer features two measurement modes, enabling conventional monitoring of the instrumental signal using the right-angle mode and a specialized mode for solid samples, known as the front-face mode. This mode allows monitoring of the emitted radiation beam at an angle of 22.5° to the excitation beam. The spectrofluorometer has been modified by incorporating an alignment system developed using 3D printing technology to position the microfluidic system within. The chip holder (FC-PRO-CH4515) is placed on the alignment system with the microreactor (FC_R150.676.2, 12 × 24 mm, 6 µLL) (Micronit, Netherlands, www.micronit.com). An electromagnet was incorporated in the optical pathway of the detector, with a pinhole that focuses the light beam. A pinhole, a small hole of 250 μm in diameter whose function is to allow the focusing of the radiation beam at the excitation wavelength directly in the reaction/detection zone of the microchannel of the microfluidic system, was added. The flow was driven by a KDS220 syringe pump (KD Scientific Inc., MA, USA, www.kdscientific.com) using 5-mL syringes (Terumo, Madrid, Spain; www.terumo.es) with poly(tetrafluoroethylene) (PTFE) tubes (inner diameter, 0.25 mm) and the appropriate PEEK connector. In addition, two Cheminert VA-CN2 injection valves (Valco Teknokroma, Barcelona, ​Spain, www.teknokroma.es) were used to inject the samples and nanoclusters, each with a 10-µL loop. All measurements were performed using the FluoroScan application software (Horiba Scientific), compatible with OriginPro 9.1.0. The data were processed using OriginPro 9.1.0 (OriginLab Co., 2013, Northampton, MA, USA) and Statgraphics Centurion 18.1.12.

### Synthesis of magnetic gold nanoclusters

Before synthesizing the magnetic gold nanoclusters, the synthesis of magnetic nanoparticles (MNPs) was carried out using the co-precipitation method described in the literature [23] in which two iron ions were precipitated using NaOH. Once the MNPs were synthesized, they were washed and centrifugated to be purified.

The synthesis of AuMNCs has been carried out using the BSA protein, which favors nucleation and stabilization, following the method described in the literature [24] with some modifications—the use of a rotavapor allowed for vigorous stirring of the mixture. At the same time, the sample is heated at 60 °C for 2 h. Two types of AuMNCs synthesis have been tested. In the first case, the precursor ions of the MNPs are added simultaneously with those of the NCs, allowing them to form simultaneously. In the second case, previously synthesized magnetic nanoparticles are added along with the precursor ions of the NCs. The second synthesis obtained the best results, so the method is carried out with this option. The process involves placing 4 mL of a 12.5 mmol L^−1^ HAuCl_4_ solution and 5 mL of a 50 mg mL^−1^ BSA solution in a round-bottom flask, both at room temperature. Subsequently, 50 µL of MNPs were added with 0.95 mL of water. After this, 50 µL of a 2 mmol L^−1^ ascorbic acid solution is added dropwise by drop in a bath at 37 °C and shaken vigorously. The flask was placed in the rotavapor at a temperature of 60 °C and, after 2 min of vigorous stirring, 0.5 mL of 1 mol L^−1^ NaOH solution was added to ensure that the synthesis was carried out at pH 12. The pH change, as indicated by a pH strip, can be observed through a gradual color change from light to dark yellow and brown, which occurs as the Au^3+^ ions are reduced to Au^+^ ions and Au^0^, respectively. The mixture is left for 2 h in the rotavapor at 60 °C. After 2 h, the finished synthesis preparation is stored at 4 °C until its later use.

### Determination of tetracyclines in microfluidic system using AuMNCs

**Fig. 1:**
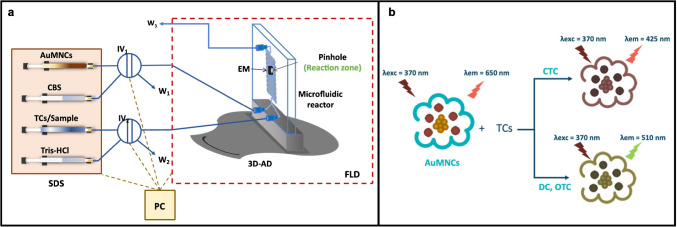
**a** Scheme of the microfluidic system used; **b** scheme of the analytical reaction between tetracyclines (TCs) and magnetic gold nanoparticles (AuMNCs). CBS: carbonate buffer solution; SDS: syringe-driven system; IV1 and IV2: injection valves; W1, W2 and W3: wastes; EM: electromagnet to immobilize AuMNCs; 3D-AD: 3D-printed alignment device; FLD: fluorometric detector; PC: personal computer as control device; CTC: chlortetracycline; DC: doxycycline; OTC: oxytetracycline

The measurements were carried out using the spectrofluorometer Fluorolog-3P, and some modifications were introduced to the microfluidic system in the sample chamber. A scheme of the complete system is shown in Fig. 1a, including the impulse system, injection valves, and the microfluidic reactor with the lab-built electromagnet device, as well as the lab-built platform for introducing the microfluidic reactor into the spectrofluorometer.

All the flows have been introduced into the system at a flow rate of 40 µL min^−1^ aided by a carbonate buffer solution (CBS) at pH 11. Ten microliters of AuMNCs is injected through an injection valve (IV_1_) to start the measurements, and after 60 s in which nanomaterials were magnetically retained in the pinhole placed in the microfluidic reactor, 10 µL of a solution of tetracyclines or samples, accordingly, is injected through another injection valve (IV_2_), the reaction occurred in the microfluidic reactor between retained AuMNCs and tetracyclines, and fluorescence intensity monitoring begins. Thirty emission spectra were taken to perform continuous measurements of the progress of the reaction for 5 min. An example of the 30 spectra register is shown in the electronic supporting materials (ESM) in Fig. S1. Once the measurement was taken, the magnetism was removed for 3 min so that the AuMNCs that had reacted with the tetracyclines and the samples were eliminated, and a new measurement could be taken with new AuMNCs.

A theoretical model of the reaction between AuMNCs and tetracyclines in the microfluidic reactor is shown in Fig. 1b. The excitation and emission spectra of AuMNCs and the formed complexes have been studied, and the procedure, along with the obtained spectra (Fig. S2), is described in ESM. The wavelengths for AuMNCs are 370 nm, the maximum excitation wavelength (λ_exc_), and 650 nm, the maximum emission wavelength (λ_em_). When the reaction with tetracyclines occurs, a change in the spectrum will be observed, depending on which tetracycline reacts. Maximum wavelengths will also be observed at 425 and 510 nm, characteristic of the binding of each tetracycline to AuMNCs. The result of the reaction leads to a decrease in the fluorescence signal corresponding to AuMNCs (650 nm) and an increase in the fluorescence signal at wavelengths characteristic of the tetracycline-AuMNCs complex (425 or 510 nm), proportional to the concentration of tetracyclines injected.

After measuring the 30 spectra to monitor the reaction between AuMNCs retained in the reaction/detection zone and tetracyclines, a representation of the injection peak along time is recorded. An example of the injection of OTC at a specific concentration is shown in Fig. S1a, as mentioned. As reflected, a specific wavelength can be selected to represent the fluorescence signal obtained over time. Therefore, the injection peak can be represented over time at various wavelengths. Thus, the peak area at specific wavelengths can be acquired to monitor the reaction of AuMNCs with tetracyclines. The analytical signal is based on the ratio between these peak areas. The ratio selected was between the peak area belonging to the interaction of AuMNCs with tetracyclines and the peak area belonging to AuMNCs (A_425_/A_650_ for CTC and A_510_/A_650_ for DC and OTC). Fig. S1b and c show how the peak corresponding to the complex of AuMNCs with tetracycline increases. In contrast, the peak corresponding to AuMNCs decreases, thus demonstrating the usefulness of using the ratio of the area of both peaks. Furthermore, since each spectrum takes about 10 s to complete, there is no need to perform any signal smoothing treatment; otherwise, too much data would be lost. Each recording was made in triplicate.

## Results and discussion

### Synthesis and characterization of magnetic gold nanoparticles

The variables involved in synthesizing AuMNCs have been studied using the univariate method. The study was conducted to determine the key features of AuMNCs, including magnetic efficiency and fluorescence emission. Table S1 in ESM summarizes the variables studied, including the range of values examined and the selected values.

Different modifications to the protocols described in the literature have been applied to the synthesis of AuMNCs, selecting the one that yields the most significant number of particles in the smaller size population, with a smaller dispersion and a higher fluorescence emission signal. For this purpose, dynamic light scattering (DLS) techniques were used to determine the size, polydispersity, Z potential, and emission spectra of AuMNCs. According to protocols, iron chlorides were introduced in the synthesis mixture to form MNPs in situ. However, the introduction of MNPs that were previously synthesized has also been considered. Table S2 in the ESM summarizes examples of some of the syntheses carried out with variations of iron chloride concentrations and the introduction of MNPs that were previously synthesized. The AuMNCs obtained by introducing iron chlorides presented a lower emission signal and a high polydispersity, as expressed in the number of populations presented, so the synthesis introducing MNPs was considered. Different amounts of MNPs were measured in the volume of the dispersion. Fig. S3 in the ESM shows the size distribution obtained when different amounts of MNPs were introduced during synthesis.

Considering the intensity obtained with DLS (Fig. S3a), a tendency towards aggregation of the AuMNCs can be observed, as indicated by the signal from larger sizes. Nevertheless, considering the size distribution of the particles, most of the observed particles had a small size, as expected for AuMNCs (Fig. S3b). The selected volume of MNP dispersion introduced was 50 µL as it presented only two populations, resulting in a smaller polydispersity (0.542 compared to 0.798 and 0.702 for another synthesis). The Z potential is a parameter that allows us to determine the stability of the dispersion and the interactions that may occur with other particles, given the surface charge of the particle. Considering the Z potential, shown in EMS in Fig. S4, all syntheses have a negatively charged surface (around − 35 mV). However, introducing 50 µL of MNPs was observed to yield a higher signal. Furthermore, when all the signals are the same, the smallest amount of MNPs was selected to minimize the introduction of significant particles. Comparing the results obtained from the emission spectra (Fig. S5 in the ESM), a higher signal was observed when 50 µL of MNPs was introduced.

**Fig. 2:**
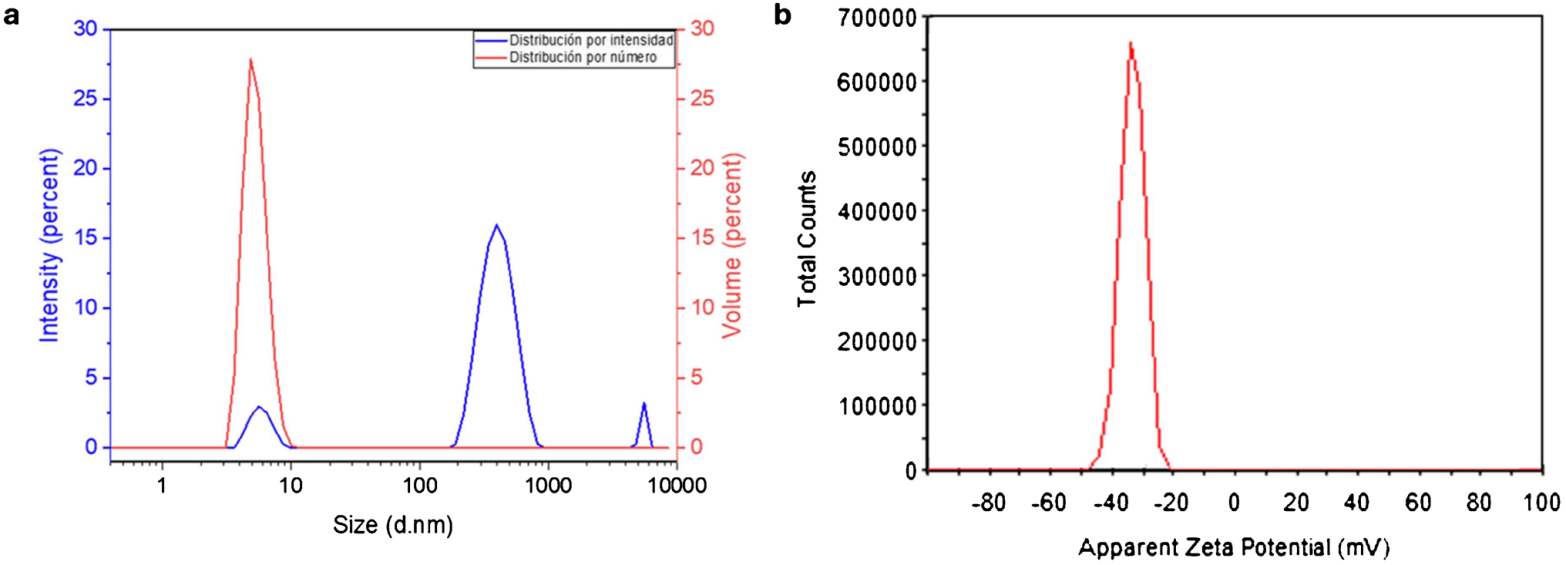
**a** Size distribution obtained with DLS of AuMNCs according to the measured signal and number of particles. **b** Z Potential of AuMNCs

Figure 2 shows the characterization of the AuMNCs. The size of the AuMNCs has been estimated using the DLS technique, yielding a hydrodynamic diameter of 6.019 ± 1.362 nm and a Z potential of − 33.5 ± 3.89 mV. As mentioned, AuMNCs tend to aggregate solutions that have been prepared in advance. In Fig. 2a, the different populations are observed in their respective sizes and the number of populations per size, illustrating the comparison. It can be concluded that, although several populations are present in this synthesis, as expected due to the formation of aggregates, the number of particles between 5 and 10 nm is significantly greater than the rest of the populations. The register of Z potential is shown in Fig. 2b. The bigger the Z potential in absolute value has the dispersion, the more stable it is, and values higher than ± 30 mV indicate the enormous stability of the dispersion, so the value found for AuMNCs indicates their stability.

### Study of the variables involved in the microfluidic system

The study of variables that may affect the hydrodynamic properties of the microfluidic system and the reaction in the reaction/detection zone is necessary to set the parameters that influence the reaction between AuMNCs and tetracyclines, thereby demonstrating the efficiency of the designed microfluidic system. The process was carried out using a univariate method, and the ratios A_425_/A_650_ and A_510_/A_650_ were determined according to the tetracycline. The studies used 50 and 200 µmol·L^−1^ concentrations of two tetracyclines, CTC and DC, to investigate the effect of each tetracycline at different emission wavelengths. Table S3 in the ESM presents the instrumental, chemical, and hydrodynamic variables; the range studied; and the selected values.

**Fig. 3:**
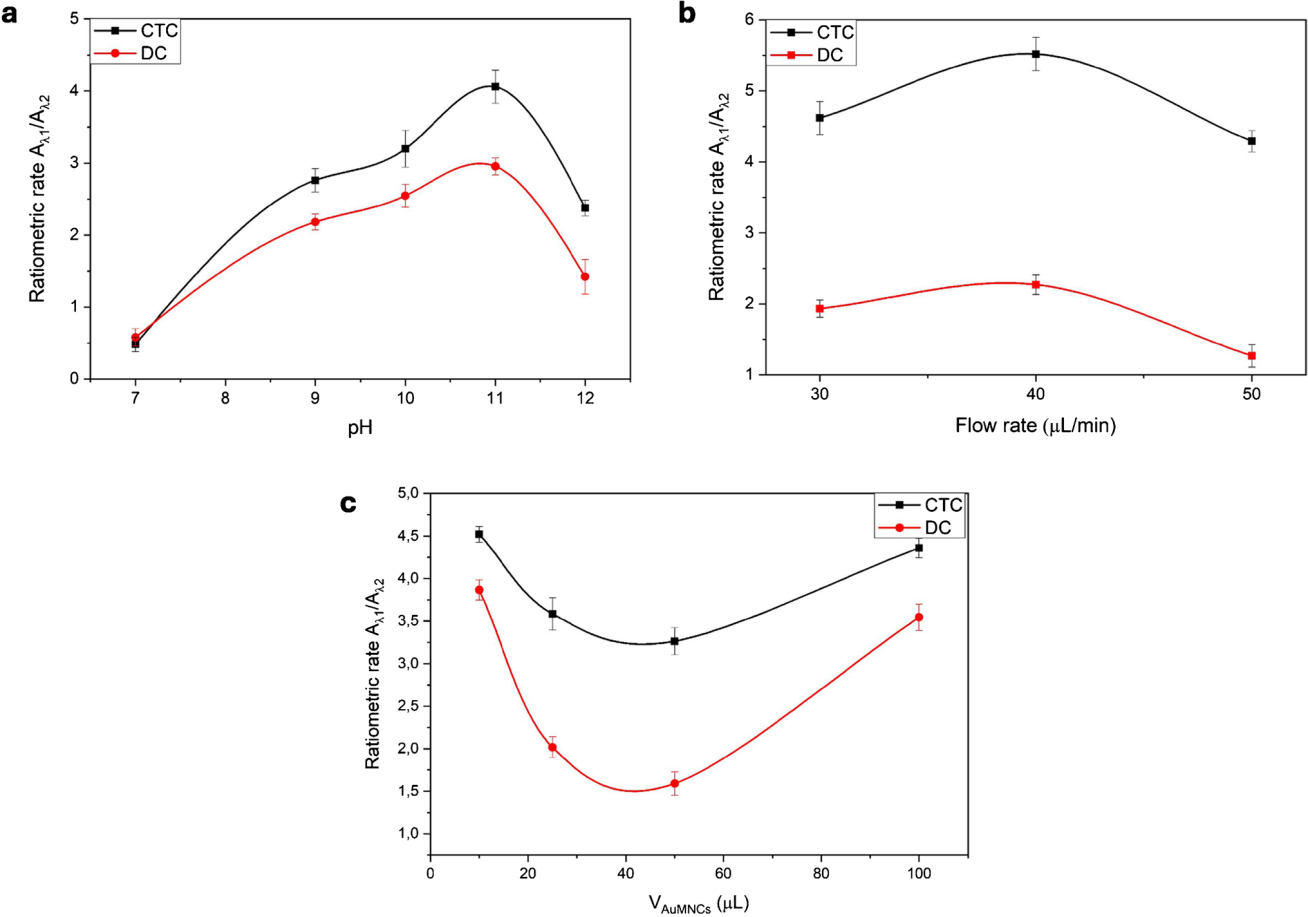
Influence of some experimental variables affecting the system and the reaction using chlortetracycline (CTC, black line) and doxycycline (DC, red line) as models: **a** pH, **b** flow rate, and **c** injection volume of AuMNCs. λ_1_ is 425 nm for CTC and 510 nm for DC; λ_2_ is always 650 nm

The study of instrumental variables has been established based on previous knowledge of spectroscopic equipment and research studies conducted within the research group regarding the reaction between AuMNCs and tetracyclines. A study of the pH influence between 7 and 12 on the emission fluorescence of AuMNCs has been carried out within the context of chemical variables. Different buffer solutions were used to test a pH range from 4 to 12: citrate buffer (pH 4–6), Tris–HCl buffer (pH 7–9), and CBS (pH 10–12). The study was planned to start at a pH 4, but the BSA protein precipitated at this pH. Figure 3a shows the influence of pH on the obtained fluorescence, with a maximum value at pH 11. The increase in the signal up to pH 11 was expected due to the partial unfolding of the BSA, exposing more functional groups to interact [25]. Higher pH values ​cause irreversible denaturation of BSA, rendering the AuMNCs unusable [26]. The buffer concentration has also been studied, obtaining the best results with 50 mmol·L^−1^. Higher concentrations could affect the reaction because the buffer could have a significant presence in the solution. CBS has less interaction with tetracyclines, but they can also interact, altering the acid-basic equilibrium of the medium and affecting the general ionic effect, which in turn impacts the colloidal stability of AuMNCs.

Among the variables affecting the microfluidic system, two different chips with reactors of 6 and 13 µL internal volume have been studied, yielding the best signal focus with 6 µL of internal volume. A pinhole has been used to focus the beam in only one reactor channel. The flow rate has been studied between 30 and 50 μL·min^−1^, and the results are shown in Fig. 3b. Lower values indicate a loss of time for sample detection. In comparison, higher values would cause overpressure and clogging problems in the microfluidic channels. These flow rates were not sufficiently high to result in an insufficient time for fluorescence collection. The best results were obtained using a flow rate of 40 μL·min^−1^.

Finally, the amount of injected and retained AuMNCs, measured in terms of the volume of solution, was studied. Different valve loops with varying injection volumes between 10 and 100 µL were assayed for this purpose. Higher values were not considered due to the large nanomaterial used and introduced in a small space. The results of this study are presented in Fig. 3c, where the maximum signal was obtained with an injection volume of 10 µL.

### Analytical features of the method

The analytical features of the developed method to determine total tetracyclines using the microfluidic system retaining magnetically AuMNCs are listed in Table S1 in ESM, including the equation parameters of the calibration graph, the limits of detection (LOD) calculated according to IUPAC recommendations [27], the linear dynamic range, and the precision expressed as a percentage of the relative standard deviation (RSD%). The calibration graphs were obtained using the ratio of the peak area at the λ_em_ of AuMNCs post- and prior-to-interaction with each tetracycline, measured at λ_exc_ 370 nm as the analytical signal, specifically A_425_/A_650_ for CTC and A_510_/A_650_ for DC and OTC.

The proposed method has been compared with the same method but without using the magnetic retention of AuMNCs to evaluate the effect of preconcentration nanomaterials in the reaction/detection zone of the microfluidic system and to demonstrate the usefulness of the magnetic retention in the developed method and the magnetic capabilities of the developed nanomaterials. The analytical features obtained are also shown in Table 1. LOD obtained with the proposed method were 0.41 µmol·L⁻^1^ for CTC, 0.70 µmol·L⁻^1^ for DC, and 0.61 µmol·L⁻^1^ for OTC. These values are notably lower than those obtained without magnetic retention, improving them in one order of magnitude, demonstrating the importance of nanomaterial preconcentration in the reaction/detection zone.

**Table 1:** Analytical features of the method with or without the use of magnetic properties of AuMNCs

		Analyte					
		CTC		DC		OTC	
Calibration graph		With retention	Without retention	With retention	Without retention	With retention	Without retention
Equation parameters^a^							
	**a (± s**_**a**_**)**	− 0.09 ± 0.01	0.34 ± 0.08	0.05 ± 2.34·10^−3^	0.03 ± 0.03	0.08 ± 1.50·10^−3^	0.09 ± 8.17·10^−3^
	**b (± s**_**b**_**)**	0.09 ± 1.55·10^−3^	0.02 ± 1.04·10^−3^	0.01 ± 3.01·10^−4^	8.55·10^−3^ ± 5.16·10^−4^	7.49·10^−3^ ± 2.02·10^−4^	2.46·10^−3^ ± 1.13·10^−4^
	***r***^**2**^	0.9962	0.9923	0.9951	0.9871	0.9949	0.9906
**LOD, µmol·L**^−**1**^		0.41	9.87	0.70	11.93	0.61	9.97
**Linear range, µmol·L**^−**1**^		1.38–200	32.92–200	2.33–200	39.77–200	2.01–200	33.21–200
**RSD%**^**b**^							
	**Min**	0.95	1.18	1.63	2.16	1.31	1.78
	**Max**	5.32	5.83	6.16	6.23	5.79	6.15

^a^The calibration plot fits an equation *y* = *a* + *b*·*x*, where *y* is the signal A_425_/A_650_ or A_510_/A_650_, respectively, and *x* is the tetracycline concentration

^b^RSD% values obtained at two concentration levels, at the LOQ (Min) and half of the linear range (Max)

Compared with previous works to determine tetracyclines using AuNCs [18, 19], the proposed method presents slightly higher LODs. However, the proposed method is label-free with simple integration and compatibility with different tetracyclines, making it a more versatile approach for general monitoring.

Furthermore, the obtained LOD are below or near the maximum residue limits (MRLs) established by regulatory authorities in different matrices. For instance, the European Union establishes MRLs of 100 µg·kg⁻^1^ (approximately 0.22 µmol·L⁻^1^ for DC and OTC) in muscle tissues [28]. Although the current method is applied to aqueous samples, it demonstrates sufficient sensitivity to be potentially adapted for MRL-compliant applications with appropriate preconcentration or extraction steps.

### Application of the method

The method was applied to determine the presence of tetracyclines in river water samples. When the method was applied to determine the analytes, no signal above the lower value of the linear range relative to tetracyclines was found. A recovery study was conducted using the addition standard method, where a concentration of 0.06 mmol·L^−1^ of each studied tetracycline was added to the samples before the SPE extraction process, aiming for a concentration of 50 µmol·L^−1^ in the extract. Table 2 summarizes the expected and found concentration of each tetracycline in samples and the calculated percentage of recovery. Observing the recovery values, most of them are acceptable according to the standard criteria, except for CTC values. This can be due to some interference present in samples influencing the signal ratio of the peak area used for this analyte.

**Table 2:** Application of the method to determine tetracyclines in river water samples

Sample	Expected concentration (µmol·L^−1^)		Found concentration (µmol·L^−1^)	% Recovery
**1**	CTC	50	43.11 ± 4.02	86.22 ± 8.04
	OTC	50	47.81 ± 2.87	95.63 ± 5.74
	DC	50	44.81 ± 3.06	89.62 ± 6.12
**2**	CTC	50	48.08 ± 2.55	96.16 ± 5.10
	OTC	50	46.62 ± 3.64	93.24 ± 7.28
	DC	50	47.15 ± 2.72	94.31 ± 5.44
**3**	CTC	50	55.35 ± 1.89	110.71 ± 3.78
	OTC	50	51.99 ± 2.59	103.97 ± 5.18
	DC	50	52.82 ± 1.76	105.63 ± 3.52

*CTC* chlortetracycline, *OTC* oxytetracycline, *DC* doxycycline

## Conclusions

In this work, a novel analytical method based on magnetically retained gold nanoclusters (AuMNCs) in a microfluidic system was successfully developed and applied for the determination of total tetracyclines, using as models three of them—chlortetracycline (CTC), doxycycline (DC), and oxytetracycline (OTC)—in water samples. The AuMNC synthesis was studied to obtain nanomaterials with strong luminescent properties and stable magnetic behavior, using BSA as a stabilizer and ascorbic acid as a reducing agent and introducing MNPs previously synthesized. Among the key parameters to carry out the determination were the reaction pH (optimal at pH 11 using carbonate buffer), the injection volume of AuMNCs (10 µL), and the flow rate within the microfluidic reactor (40 µL·min⁻^1^).

The developed system exhibits limits of detection of 0.41 µmol·L⁻^1^ (CTC), 0.70 µmol·L⁻^1^ (DC), and 0.61 µmol·L⁻^1^ (OTC). These values were up to one order of magnitude lower than those obtained without magnetic retention, confirming the benefit of nanomaterial preconcentration in the detection zone. The method showed good linearity, precision, and recoveries between 86 and 111% in river water samples after pre-treatment. When compared to previously published methods, including specific ratiometric fluorescent sensors, the proposed approach offers a versatile, label-free, and simpler alternative, suitable for real-time environmental monitoring. The method has a limitation to discriminate some tetracyclines, as they modify the same emission wavelength of AuMNCs.

Future research should focus on adapting this microfluidic detection system for complex matrices, such as food extracts or biological fluids, by incorporating appropriate sample pretreatment steps. Additionally, the platform could be extended to detect other classes of contaminants by functionalizing the AuMNCs with selective ligands or aptamers and integrating multiple detection zones for multiplexed analysis in a lab-on-a-chip format.

## Supplementary information

Below is the link to the electronic supplementary material.

Supplementary File 1 (DOCX 4.79 MB)

## Data Availability

Data is provided within the manuscript or supplementary information files.
