# Supplementary material for: Application of magneto-luminescent gold nanoclusters with microfluidic systems to the determination analysis of tetracyclines
Source: Mikrochim Acta. 2025 Jul 31;192(8):545. doi: 10.1007/s00604-025-07405-5 (PMC12313782; doi:10.1007/s00604-025-07405-5)
Supplement: Supplementary file 1 — (DOCX 4.79 MB) [file 604_2025_7405_MOESM1_ESM.docx]

**TITLE:** Application of magneto-luminescent gold nanoclusters with microfluidic systems to the analysis of tetracyclines

**AUTHORS:** Javier Palma Roldán, Vanesa Román-Pizarro, Miguel Ángel García-Granados, Juan Manuel Fernández-Romero, Ángela Écija-Arenas

Departamento de Química Analítica, Instituto Universitario de Investigación en Química Fina y Nanoquímica (IUNAN), Universidad de Córdoba, Campus de Rabanales, Edificio Anexo “Marie Curie”, E-14071 Córdoba, España

First author: Javier Palma-Roldán

E-mail address: t02paroj@uco.es

* Corresponding author: Ángela Écija-Arenas

ORCID: 0000-0003-3240-5769

E-mail address: q92ecara@uco.es

Other authors: Vanesa Román-Pizarro

ORCID: 0000-0003-3240-5769

E-mail address: q52ropiv@uco.es

Miguel Ángel García-Granados

E-mail address: a62gagrm@uco.es

Juan Manuel Fernández-Romero

ORCID: 0000-0001-8443-1358

E-mail address: qa1feroj@uco.es

**Materials and methods**

**Determination of tetracyclines in microfluidic system using AuMNCs**


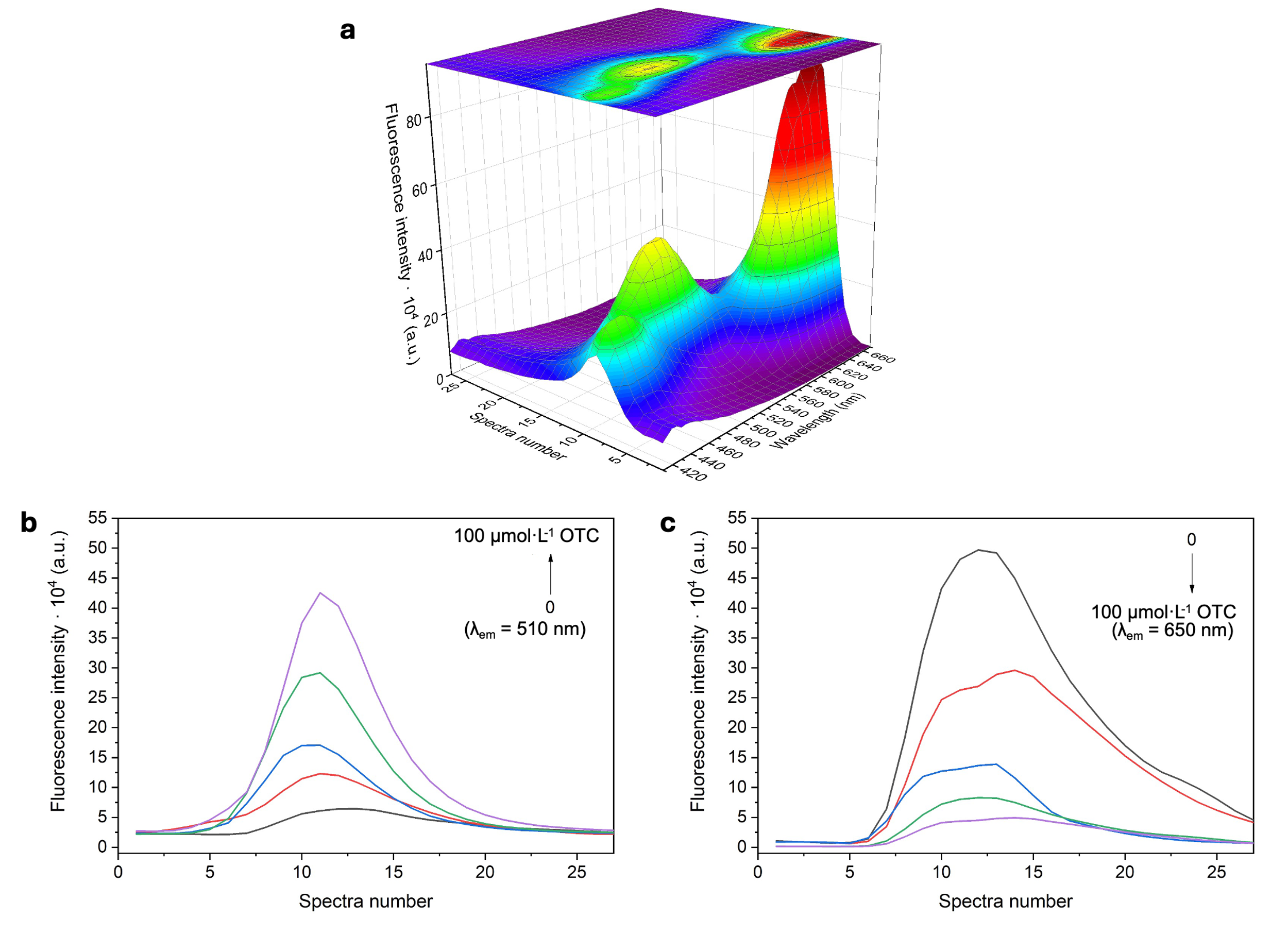


**Fig. S1 a** Example of a recording of 30 emission spectra (λexc = 370 nm) between 410 and 670 nm of the reaction to perform continuous measurement of the reaction progress for 5 min between AuMNCs magnetically retained in the reaction/detection zone of the microfluidic reactor and oxytetracycline (OTC) injected at a specific concentration **b** Decrease of the injection peak at 510 nm when increasing concentrations of injected OTC **c** Increase of the injection peak at 650 nm when increasing concentrations of injected OTC

***Excitation and emission spectra of AuMNCs and the formed complexed with tetracyclines***

The fluorescence emission after the formation of the AuMNCs and their reaction with the different tetracyclines has been monitored through emission spectra measurements over time. For that, the excitation and emission spectra of these complexes have been studied in batches using a 96-well plate placed in an auxiliary module (Cary Eclipse Microplate reader from Agilent Technologies) of the Agilent Cary Eclipse spectrofluorometer (Walnut Creek, CA, USA, http://www.varianinc.com). The specific software package for the Agilent Cary Eclipse spectrofluorometer was used to process the measurements. The excitation and emission spectra are determined with an excitation and emission slit width of 10 nm and a 600 V photomultiplier tube power for the excitation spectrum and 800 V for the emission spectrum. The emission spectrum (λ_exc_ = 370 nm) was monitored from 410 nm to 670 nm to observe the changes in fluorescence intensity at 510 nm (λ_em_ of the union of oxytetracycline or doxycycline with AuMNCs), at 425 nm (λ_em_ of chlortetracycline with AuMNCs) and 650 nm (λ_em_ of the AuMNCs).

Solutions of AuMNCs were introduced into the wells separately (50 μL of AuMNCs with 100 μL of Tris-HCl buffer); 50 µmol L^-1^ of tetracycline separately (50 μL of each tetracycline 0.3 mmol L^-1^ in 100 μL of Tris-HCl buffer); and the mixture of AuMNCs with 50 µmol L^-1^ of tetracycline (50 μL of AuMNCs and 50 μL of each tetracycline 0.3 mmol L^-1^ with 50 μL of Tris-HCl buffer). **Fig. S2a** shows the excitation spectrum of the AuMNCs separately, whose maximum λ_exc_ value corresponds to 370 nm. As shown in **Fig. S2b**, the λ_em_ of the AuMNCs corresponds to 650 nm. The interaction of AuMNCs with chlortetracycline (CTC) causes a significant increase in fluorescence at 425 nm. The interaction of AuMNCs with oxytetracycline (OTC) and doxycycline (DC) causes a substantial increase in fluorescence at 510 nm, being higher in the case of DC. Regarding the intensity of fluorescence emitted by the tetracyclines without interacting, a very low signal is observed compared to that emitted after the interaction with the AuMNCs. The change in fluorescence is also shown in **Fig. S2c**, where the change in fluorescent emission can be observed when placing the solutions of AuMNCs alone and with tetracyclines under an ultraviolet light lamp.


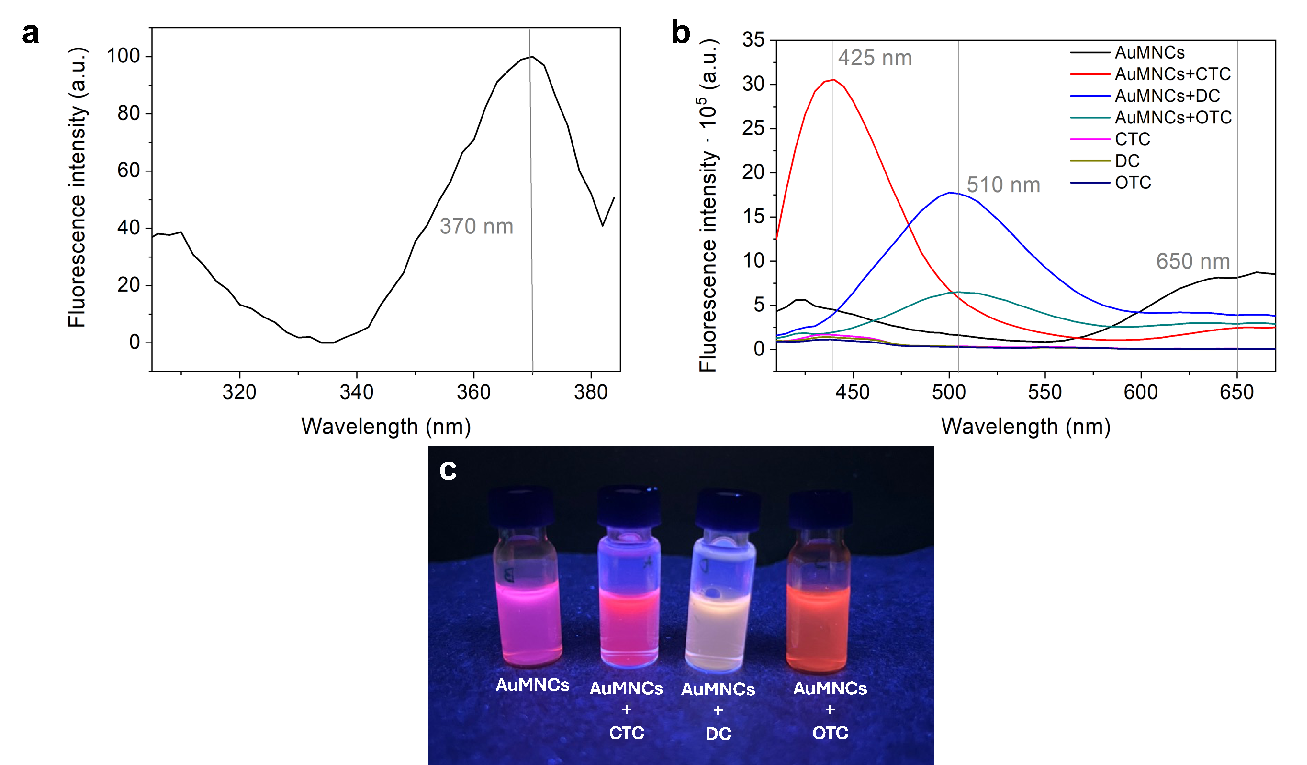


**Fig. S2 a** AuMNCs excitation spectrum **b** Emission spectra of: AuMNCs, tetracyclines after the interaction with the AuMNCs (AuMNCs + chlortetracycline (CTC), AuMNCs + doxycycline (DC) and AuMNCs + oxytetracycline (OTC)) and the tetracyclines without interaction (CTC, DC and OTC) **c** Image showing the fluorescence of the AuMNCs and the color change when adding the tetracyclines

**Results and discussion**

**Synthesis and characterization of magnetic gold nanoparticles (AuMNCs)**

**Table S1** Study of the variables affecting the AuMNCs synthesis

| **Type of variable** | **Variable** | **Range studied** | **Optimal value** |
| --- | --- | --- | --- |
| Chemical variables | [HAuCl_4_], mmol·L^-1^ | 10 – 15 | 12.5 |
|  | [BSA] mg·mL^-1^ | 20 – 60 | 50 |
|  | [C_6_H_8_O_6_], mmol·L^-1^ | 1 – 5 | 2 |
|  | [NaOH] mol·L^-1^ | 0.5 – 2 | 1 |
|  | FeCl_2_, μL | 250 – 500 | 250 ^a^ |
|  | FeCl_3_, μL | 250 – 500 | 250 ^a^ |
|  | MNPs, μL | 50 – 500 | 50 |
|  | pH | 6 – 11 | 10 |
| Physical variables | Temperature, °C | 25 – 60 | 60 |
|  | Time, h | 1 – 12 | > 2, < 4 |
| ^a^ The optimal values for both iron chlorides correspond to 250 µL each one; however, iron chlorides were substituted by MNPs in the final synthesis of AuMNCs due to the increase in the fluorescence signal obtained | | | |

**Table S2** Some of the modifications considered during the synthesis process

| **Synthesis** | **Modifications considered** |
| --- | --- |
| 1 | 500 μL FeCl_2_ 5mM and 500 μL FeCl_3_ 5 mM |
| 2 | 250 μL FeCl_2_ 5 mM, 250 μL FeCl_3_ 5 mM and 500 μL H_2_O |
| 3 | 500 μL FeCl_2_ 20 mM and 500 μL FeCl_3_ 20 mM |
| 4 | 250 μL MNPs and 750 μL H_2_O |
| 5 | 50 µL MNPs and 950 µL H_2_O |
| 6 | 500 μL MNPs and 500 μL H_2_O |


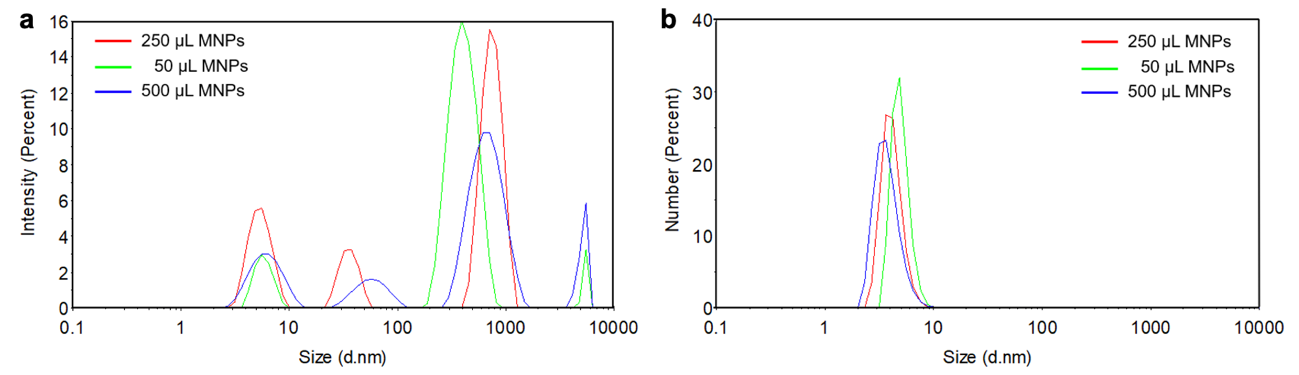


**Fig. S3** Size distribution of AuMNCs synthesized with different amounts of MNPs according to measured intensity (**a**) and according to the number of particles (**b**)


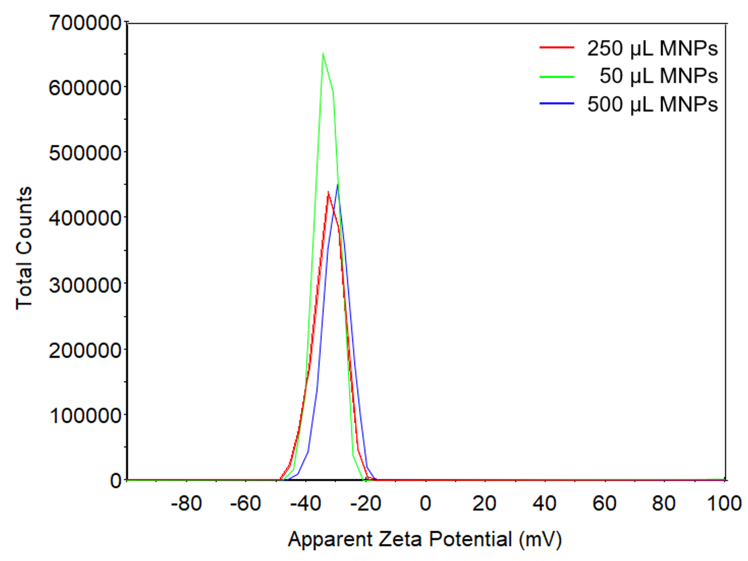


**Fig. S4** Z potential of AuMNCs synthesized with different amounts of MNPs


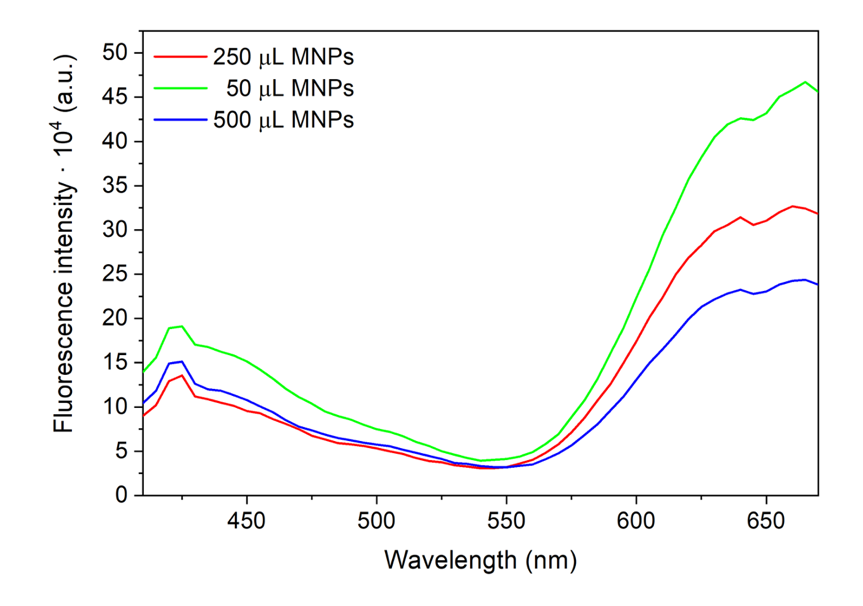


**Fig. S5** Emission spectra of AuMNCs synthesis using different amounts of MNPs

**Study of the variables involved in the microfluidic system**

**Table S3** Study of the variables affecting the system

| **Type of variable** | **Variable** | **Range studied** | **Optimal value** |
| --- | --- | --- | --- |
| Instrumental variables | λ_exc_, nm | 200-800 | 370 |
|  | λ_em_, nm | 200-800 | 425, 510 and 650 |
|  | Excitation/emission slit | 1-10 | 2/5 |
|  | PMT gain, V | 800-950 | 950 |
| Chemical variables | [Buffer Tris-HCl], mmol·L^-1^ | 10-100 | 50 |
|  | pH | 4-12 | 11 |
|  | Rotation angle ° | 20-90 | 75 |
| Microfluidic variables | Pinole diameter, µm | - | 250 |
|  | Reactor volume, µL | 6-13 | 6 |
|  | Flow rate, μL·min^-1^ | 30-50 | 40 |
|  | V_Injection_ AuMNCs, μL | 10-100 | 10 |
|  | V_Injection_ TCs/Samples, μL | 10-100 | 10 |
|  | Retention time, min | 0-5 | 1 |
|  | Register time, cycles | 20-50 | 25 |
|  | Washing time, min | 0-5 | 3 |
